# A Step in the Right Direction

**Published:** 1872-02

**Authors:** 


### A STEP IN THE RIGHT DIRECTION.

We are pleased to chronicle the organization of the Middle Georgia Medical Society, and hope to see the example imitated by the physicians throughout the State. We shall look to this and other local organizations as the only really reliable and feasible means by which the profession is to be protected from irregularities in its ranks. It is only in small bodies and in circumscribed localities that discipline can be administered. Besides, for obvious reasons, the local societies do infinitely more for the advancement of medical science, by frequent meetings and systematic investigations, which are, to a great extent, incompatible with the larger organizations, which naturally assume the character of social reunions.

